# Association of Glial Activation and α-Synuclein Pathology in Parkinson’s Disease

**DOI:** 10.1007/s12264-022-00957-z

**Published:** 2022-10-14

**Authors:** Rui Wang, Haigang Ren, Elena Kaznacheyeva, Xiaojun Lu, Guanghui Wang

**Affiliations:** 1 Center of Translational Medicine, First People’s Hospital of Taicang, Taicang Affiliated Hospital of Soochow University, Suzhou, 215400 China; 2 Laboratory of Molecular Neuropathology, Jiangsu Key Laboratory of Neuropsychiatric Diseases and College of Pharmaceutical Sciences, Soochow University, Suzhou, 215123 China; 3 Institute of Cytology, Russian Academy of Sciences, Saint-Petersburg, Russia 194064; 4 Department of Neurosurgery, First People’s Hospital of Taicang, Taicang Affiliated Hospital of Soochow University, Suzhou, 215400 China

**Keywords:** Parkinson's disease, α-synuclein pathology, Microglial activation, Astrocyte activation, Neuroinflammation

## Abstract

The accumulation of pathological α-synuclein (α-syn) in the central nervous system and the progressive loss of dopaminergic neurons in the substantia nigra pars compacta are the neuropathological features of Parkinson's disease (PD). Recently, the findings of prion-like transmission of α-syn pathology have expanded our understanding of the region-specific distribution of α-syn in PD patients. Accumulating evidence suggests that α-syn aggregates are released from neurons and endocytosed by glial cells, which contributes to the clearance of α-syn. However, the activation of glial cells by α-syn species produces pro-inflammatory factors that decrease the uptake of α-syn aggregates by glial cells and promote the transmission of α-syn between neurons, which promotes the spread of α-syn pathology. In this article, we provide an overview of current knowledge on the role of glia and α-syn pathology in PD pathogenesis, highlighting the relationships between glial responses and the spread of α-syn pathology.

## Introduction

Parkinson’s disease (PD) is the second most common age-related neurodegenerative disease following Alzheimer’s disease (AD), characterized by motor symptoms of bradykinesia, rigidity, resting tremor, and postural instability as well as non-motor symptoms of constipation, depression, sleep disorders, and cognitive decline [1]. The onset of disease generally occurs in individuals >60 years old, and it is estimated that ~10 million people worldwide have PD [2–4]. Currently, there are no effective therapies to block the progression of PD [5]. Although 5%–10% of PD cases are familial, the majority of cases are sporadic [3, 6]. The hallmarks of pathological changes in PD are the loss of dopaminergic (DA) neurons in the substantia nigra pars compacta (SNpc) and the deposition of α-synuclein (α-syn) in Lewy bodies (LBs) and neurites (LNs) that are widely distributed in the brains of PD cases [6–9]. The LBs are composed of phosphorylated α-syn (S129), ubiquitinated proteins, and other damaged organelle components [10–12]. Pathological α-syn can recruit and convert unfolded α-syn to form pathological amyloid fibrils in neurons. The fibrils undergo fragmentation to form small fragments and oligomers that are secreted from the neuron. The oligomers then enter the next neuron through receptor-mediated endocytosis, leading to the spread of pathological α-syn throughout the brain in a prion-like manner [13–15]. In diseased PD brains, glial activation and the accumulation of α-syn in neurons are often accompanied by LB and LN pathology and neurodegeneration [16–19]. Although it is accepted that glial activation is a response to neuronal damage by misfolded α-syn that is toxic to neurons, recent studies suggest that the reaction of glia may be more than just a passive response; it may contribute to the spread of α-syn pathology and the development of PD-related pathology [19, 20].

In this review, we discuss the association of glial activation and α-syn pathology in PD. We summarize the studies on the activation of glial cells by α-syn species to discuss the response of glial cells to α-syn aggregates. We also described the roles of glial cells in the clearance and transmission of α-syn aggregates that contribute to the spread of α-syn pathology.

## α-Syn Pathology

### α-Syn Aggregation and Toxicity

The α-syn protein is encoded by the *SNCA* gene, and has an average molecular weight of ~14 kDa [21]. α-Syn protein is mainly located at the presynaptic terminal in the central nervous system (CNS) and is involved in the release of synaptic vesicles by promoting soluble NSF attachment protein receptor (SNARE)-complex assembly [22, 23]. α-Syn natively exists as soluble compact monomers or α-helically folded tetramers to avoid aggregation [13, 14]. However, under some pathological conditions, α-syn monomers may be misfolded to form soluble pathological oligomers, or insoluble β-sheet-rich fibrils [24–26].

α-Syn oligomers are more toxic than other assemblies (monomers, fibrils, or aggregates) [27]. The point mutation of α-syn with E35K, E57K, or E46K is prone to oligomerization and leads to neuronal damage [27–29]. In lentivirus-infected brains, both wild-type (WT) α-syn and the E57K mutant form SDS-insoluble α-syn oligomers that are detected in the membrane fractions, suggesting that α-syn oligomers interact with lipid membranes. However, more oligomers are formed by α-syn E57K than WT α-syn. α-Syn E57K prefers to form oligomers, but WT α-syn rapidly forms fibrils from oligomers. Moreover, more DA neuronal loss has been reported in mice infected with E57K mutant α-syn than with WT α-syn, further suggesting toxic effects of α-syn oligomers on DA neurons [27]. In induced pluripotent stem cell (iPSC)-derived neurons, overexpression of α-syn with the familial mutation E46K or an artificially-induced E57K mutation results in more α-syn oligomers than those expressing WT α-syn. Moreover, α-syn E46K or E57K mutation leads to abnormalities in axonal and synaptic integrity due to the abnormal distribution of motor factors for the anterograde axonal transport of mitochondria [29]. In addition, transgenic mice that harbor the E57K mutation show evident synaptic abnormalities and neuronal loss, with learning and memory defects [28], suggesting that oligomeric α-syn plays crucial roles in α-syn-induced PD pathology.

**Fig. 1:**
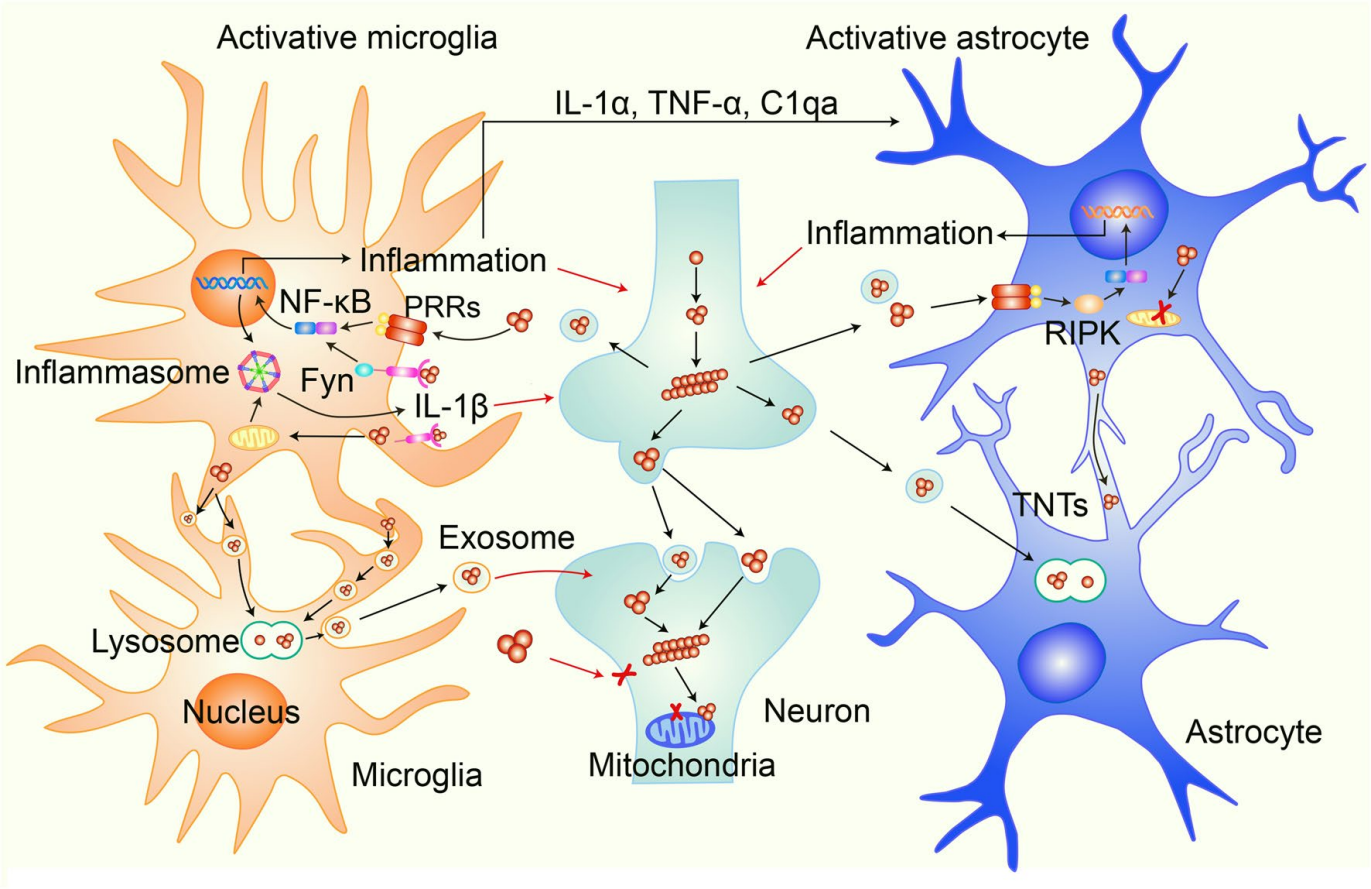
Schematic of the impact of α-syn aggregates on the activation of microglia and astrocytes and their contributions to α-syn pathology. α-Syn aggregates propagate between neurons and are released from them. Extracellular α-syn aggregates activate microglia by initiating the NF-κB-dependent inflammatory response and inflammasome activation *via* pattern recognition receptors (PPRs) or Fyn. Extracellular α-syn aggregates induce the NF-κB-dependent inflammatory response in astrocytes, and this is associated with the activation of RIPK signaling. Meanwhile, microglia phagocytose extracellular α-syn aggregates and transport them to lysosomes for degradation. Overloaded α-syn in microglia or astrocytes can be transmitted between microglia through cellular networks or between astrocytes through tunneling nanotubes (TNTs). In addition, microglia and astrocytes activated by α-syn aggregates produce pro-inflammatory factors, which damage neurons and promote the transmission of α-syn pathology between neurons.

The cell membranes and mitochondria are the most common targets of exogenous α-syn oligomers in various types of neuron [30–35]. α-Syn oligomers have a high affinity for the lipid bilayer of the cell and organelle membranes, resulting in a disruption of membrane integrity [32–34]. The high affinity of the oligomers for cell membranes also determines their ability to enter neurons, which may be superior to other forms of α-syn [34]. After entering neurons, α-syn oligomers target and accumulate in the outer or inner membrane of mitochondria [33, 36, 37]. It has been reported that α-syn oligomers interact with the translocase of the outer mitochondrial membrane 20 (TOM20), resulting in an inhibition of mitochondrial protein import [36, 38]. Moreover, α-syn oligomers impair the respiratory chain complexes to decrease ATP production and increase oxidative stress when they are translocated to the mitochondrial inner membrane [37]. Interestingly, neutralizing α-syn oligomers with the A11 antibody, which is a polyclonal antibody against oligomers, blocks α-syn oligomers from entering cells and attenuates oligomer toxicity [34], further suggesting a role for α-syn oligomers in neuronal toxicity. Thus, the conformational changes of α-syn under pathological conditions induce α-syn misfolding and aggregation, leading to the formation of α-syn oligomers and fibrils [34]. Blocking oligomer entry into cells might be a promising strategy to inhibit the toxicity of α-syn aggregation (Fig. 1).

### Pathological α-syn Transmission

The hypothesis of α-syn transmission was first proposed by Braak and colleagues [39]. Their study showed that the distribution of α-syn pathology in the brain is correlated with the severity of PD symptoms. At the early stage of PD, α-syn pathology often appears in the olfactory nucleus and olfactory bulb, the lower brain stem, and the dorsal IX/X motor nuclei [39, 40]. With the progress of disease, α-syn pathology is observed in the midbrain at the developing stage and in the neocortex at the last stage. Based on these findings, it has been speculated that α-syn pathology is initiated in the lower brain stem, spreads through neural interconnections, and eventually reaches the neocortex [39].

More convincing evidence that suggests α-syn transmission between cells came from clinical findings in 2008 [41, 42]. α-Syn- and ubiquitin-positive LBs and LNs have been found in transplanted DA neurons in the grafts from PD patients who died within 11–16 years after bilateral transplantation with human fetal mesencephalic tissue, suggesting that the α-syn pathology in grafted DA neurons comes from the propagation of pathological α-syn from surrounding host neurons [41, 42].

In recent years, various *in vitro* and *in vivo* models of α-syn transmission have been established to confirm the α-syn-spreading hypothesis based on findings from PD patients [43–47]. It is well accepted that the transmission of α-syn is due to the prion-like properties of its misfolded form, similar to other pathogenic proteins in neurodegenerative diseases [25, 26]. Prions are composed of misfolded prion proteins (PrPs) that can infect the host and force the native normal structure of PrPs to form an anomalous misfolded structure and aggregate in host cells. Once the aggregates reach a certain extent, they undergo fragmentation to form new templates (seeds), thus initiating a chain-reaction of PrP misfolding and aggregation [25]. Without misfolded α-syn, the transfer of WT α-syn between cells does not induce α-syn pathology. However, like prions, misfolded α-syn is prone to aggregation, which induces the structural change of normal α-syn monomers that are recruited to α-syn aggregates and form pathological forms, ranging from small oligomers to large β-sheet-rich fibrils [25, 48]. Under certain conditions, these fibrils undergo fragmentation to generate new small aggregates called seeds to propagate the pathogenic form of α-syn, which results in the spread of α-syn pathology from cell-to-cell and brain region-to-brain regions when α-syn seeds are released from one cell and taken up by another [25, 26].

α-Syn monomers can be induced to form misfolded α-syn and then β-sheet-rich fibrils *in vitro* [49]. Smaller pre-formed fibrils (PFFs) are obtained by ultrasonic fragmentation of fibrils [49]. The introduction of PFFs into human cell lines by liposomes induces overexpressed WT α-syn to form amyloid-like fibrils that are highly ubiquitinated and phosphorylated, sharing properties with those in PD patients [43, 50]. In mouse primary neurons, PFFs induce endogenous α-syn to form LB/LN-like structures in a time-dependent manner, resulting in synaptic dysfunction and neuronal death. In addition, the severity of the defects in neuronal network activity matches the development of α-syn pathology, similar to the progression of α-syn pathology in PD patients [44]. In animals, a single brain site injection with recombinant PFFs or brain homogenates containing pathological α-syn assemblies into A53T transgenic mice that overexpress the human *SNCA* gene with the familial A53T mutation induces the development of α-syn pathology in extensive areas of the brain. This confirmed the α-syn transmission hypothesis for the first time *in vivo* [46]. In addition, intrastriatal inoculation with recombinant PFFs in WT mice also induces phospho-α-syn-positive LB/LN aggregates from endogenous α-syn, showing the distribution of α-syn pathology along with interneuronal connectivity in the brain. These mice also show time-dependent α-syn pathology, neuronal loss, and motor defects similar to sporadic PD-like pathology [51]. Moreover, fewer motor defects and α-syn pathology have been reported in *Snca*+/– heterozygous mice, further suggesting the involvement of WT α-syn in α-syn pathology after PFF treatment [51]. Therefore, the PFF mouse model has been widely used in PD studies. Most interestingly, in addition to the transmission of α-syn pathology in the CNS, peripheral PFF inoculation also induces α-syn pathology in the CNS [52, 53]. Inoculation of PFFs into the gastroduodenal tract in WT mice induces α-syn pathology in multiple brain regions, including the dorsal motor nucleus, brainstem, midbrain, and cortex [53]. The mice present olfactory dysfunction, motor deficits, and cognitive decline, in which the phenotypes are similar to the clinical symptoms of PD patients, indicating that the abnormal aggregation of α-syn in the gastroduodenal tract can initiate the transmission of α-syn pathology from the gut to the CNS and affect neuronal functions in multiple brain regions [53]. In addition, α-syn pathology is present in multiple brain regions, starting from the dorsal motor nucleus of the vagus in the medulla and locus coeruleus in the pons 1 month after the injection of PFFs into the muscle layers of the pylorus and duodenum, and then spreads to the amygdala, SNpc, striatum, hippocampus, and cortex [53]. Loss of DA neurons and defects in motor behavior have also been reported in mice that receive a PFF injection into the pylorus and duodenum. Importantly, truncal vagotomy or knockout of the *Snca* gene blocks the transmission of α-syn pathology from the gut to the CNS and prevents DA neuronal loss and motor deficits, showing that the vagus nerve is necessary for the transmission of α-syn pathology from the peripheral nervous system (PNS) to the CNS and that WT α-syn progresses into pathogenic α-syn, contributing to the spread of α-syn pathology [53] (Fig. 1).

Both *in vitro* and *in vivo* data support the Braak hypothesis that α-syn is transmitted from cell to cell in a prion-like manner. The presence of LB/LN pathology in multiple brain regions in PD patients may be a result of α-syn transmission in a time- and region-dependent manner. The toxicity of pathological α-syn, with a wide distribution and spread of toxic α-syn species to multiple regions in the CNS and PNS, may cause dysfunction of neurons, which is responsible for the motor and non-motor symptoms in PD patients.

## Microglia and α-Syn Pathology

Microglia are resident myeloid macrophages in the CNS that play important roles in maintaining neuronal homeostasis by the pruning of synapses, elimination of protein aggregates and dead cells, maintenance of synaptic plasticity, and immune monitoring in CNS development and diseases [54, 55]. Microglia express a large number of pattern-recognition receptors, such as toll-like receptors (TLRs), to recognize damage-associated molecular patterns (DAMPs) and pathogen-associated molecular patterns (PAMPs), which play roles in the immune response [55]. Activated microglia can internalize pathogenic species and degrade them through various endocytic pathways. Activated microglia also increase the expression of relevant inflammatory modules, including chemokines and interferons, which are major components in neuroinflammation [55, 56]. The increased levels of inflammatory factors in PD patients and animal models indicate that microglial activation plays a key role in PD pathogenesis [57–59]. Inhibition of microglial activation by MCC950, a small molecule that inhibits inflammasomes by blocking the activation of NLR family pyrin domain containing 3 (NLRP3), or by NLY01, a glucagon-like peptide-1 receptor agonist that inhibits microglial activation, decreases PFF-induced α-syn pathology and DA neuronal loss as well as motor behavior deficits in a PFF mouse model in which a single striatal injection of synthetic α-syn fibrils drives the transmission of pathological misfolded α-syn in WT mice [60, 61], which suggests a link between microglial activation and α-syn pathology in PD.

### Induction of Microglia-associated Neuroinflammation by Different α-Syn Species

A growing body of evidence indicates that various receptors on the surface of microglia are involved in binding to different α-syn species to mediate neuroinflammation, causing augmentation of neurotoxicity by the release of different inflammatory cytokines that affect neurons in different cell or animal models [62–66] (Table 1). Kim and colleagues first reported that α-syn oligomers secreted by neuronal cells are endogenous TLR2 receptor agonists for microglial activation [62]. After treatment with conditioned medium from differentiated SH-SY5Y cells in which human WT α-syn is overexpressed, primary microglia can be activated, as evidenced by morphological change to an amoeboid shapes, the production of pro-inflammatory cytokines, and an increase in proliferation [62]. Transcriptome analyses suggest the involvement of the TLR and Jak-STAT signaling pathways in α-syn oligomer-induced inflammatory activation. Blocking TLR2 signaling by knocking out the *Tlr2* gene in microglia or applying anti-TLR2 antibodies inhibits inflammatory activation. The conditioned medium from SH-SY5Y cells that overexpress α-syn mainly contains β-sheet-rich oligomers of α-syn but also monomers. Both α-syn monomers and oligomers derived from SH-SY5Y cells induce microglial activation, but the effects of monomers are weak. Injection of these oligomers into the cerebral cortex induces inflammatory activation in WT mice but not in *Tlr2*-knockout mice [62]. Thus, the data suggest that TLR2 is an effective agonist responsible for α-syn-induced microglial activation.

**Table 1:** Different receptors respond to diverse α-syn forms to initiate microglial activation

Receptor	Signaling	Syn forms	References
TLR1/2	TLR1/2-MyD88-NF-κB & inflammasome priming	Monomers, oligomers & fibrils	[62, 64, 65, 95, 109]
TLR-4	TLR1/2-MyD88-NF-κB	Monomers, oligomers & fibrils	[63, 110]
TLR5	Inflammasome activation	Monomers & oligomers	[64]
CD36	CD36-Fyn-PKCδ-NF-κB & inflammasome activation	Monomers & fibrils	[65, 111]
FcγR	FcγR-NF-κB	Fibrils	[112]

In addition to TLR2, TLR4 can also recognize oligomeric α-syn and initiate the TLR4-MyD88 signaling pathway, thereby activating transcription and releasing inflammatory factors. Moreover, in comparison to TLR2, TLR4 has better selectivity for the α-syn oligomer and mediates a stronger inflammatory response. The use of the TLR4 inhibitors RSLA and TAK242 or the molecular chaperone clusterin that binds to oligomeric α-syn blocks the microglial activation induced by α-syn oligomers in BV2 microglial cells [63]. In addition to TLR2, α-syn monomers and oligomers also bind to TLR5 to promote inflammasome assembly and activate the inflammatory response [64]. The different α-syn species bind to various TLRs and have differential activities in NLRP3 inflammasome activation in lipopolysaccharide (LPS)-primed primary microglia [64].

In addition to the classical TLR pathway, Fyn, a non-receptor Src family tyrosine kinase, mediates aggregated α-syn PFF uptake and NLRP3 inflammasome activation [65]. The aggregated α-syn PFF produced by *in vitro* incubation of human recombinant α-syn activates CD36, which recruits and activates Fyn, further inducing a PKCδ-dependent NF-κB signaling pathway. Infection with AAV-α-syn induces microglial activation in the SNpc in WT mice but not in *Fyn*-knockout mice [65]. The activation of microglia by aggregated α-syn also increases the toxic effects of α-syn on DA neurons [67]. In a mouse model, overexpression of human α-syn by the injection of AAV-α-syn into the SN induces the activation of NF-κB two weeks after injection in WT mice but not in mice in which the family of Fc gamma receptors (FcγRs) is deficient [68]. In addition, the activation of microglia and the degeneration of DA neurons induced by overexpression of α-syn are attenuated in FcγR–/– mice. As FcγR is expressed on the surface of microglia but not neurons in the CNS, the attenuation of DA neuronal loss suggests that the activation of microglia contributes to α-syn-induced neurodegeneration [68].

Pattern recognition receptors (PPRs) on microglial membranes, including TLR2, TLR4, and TLR5, as well as non-receptor-dependent kinases play a crucial role in α-syn-induced inflammatory activation. PPRs, along with inflammasomes, sense PAMPs and DAMPs upon neuronal damage. In PD brains, PPRs are strongly expressed and closely associated with microglial activation [62–66]. Recently, inflammasomes have received great attention as contributors to α-syn-induced neuroinflammation [61, 64, 65, 69]. The inflammasome in immune cells is a multiprotein complex that regulates inflammatory responses by sensing PAMPs or cellular stress [70]. Dysfunction of inflammasomes is associated with autoimmune diseases, neurodegenerative diseases, and cancers [70]. The most important type of inflammation is the NLRP3 inflammasome, which was first shown to be involved in cryopyrin-associated periodic α-syndrome [71]. The NLRP3 inflammasome is assembled by the sensor element NLRP3, the adaptor element apoptosis-associated speck-like protein (ASC), and the effector element caspase. Upon stimulation, NLRP3 oligomerizes and recruits ACS through the amino-terminal pyrin domain, which in turn recruits caspase-1 through the carboxy-terminal caspase recruitment domain. Caspase-1 is a protease that is able to cleave the precursor forms of interleukin-1β (IL-1β) and IL-18, producing mature IL-1β and IL-18 that are secreted from cells and initiate inflammatory responses. Activation of the NLRP3 inflammasome is usually considered to be a two-step process: priming and activation. Priming involves sensing PAMPS or DAMPs by PPRs to initiate NF-κB signaling, leading to an increase in the transcription of inflammatory factors and inflammasome components. In the activation stage, NLRP3 senses various stimuli, including bacteria, viruses, ATP, or cell stresses such as mitochondrial oxidative stress, followed by the assembly of inflammasomes, leading to the processing of precursor inflammatory cytokines and the release of inflammatory cytokines from cells [71]. Neuroinflammation mediated by the NLRP3 inflammasome has been widely reported in AD [71–73]. Pathological Aβ aggregates activate microglia to induce the assembly of inflammasomes and the release of IL-1, subsequently damaging neurons [72]. Moreover, IL-1, by binding to IL-1R on neurons, increases the phosphorylation and aggregation of Tau through the activation of tau-associated kinase and phosphatase signaling pathways, contributing to the Tau pathology in AD [73].

α-Syn aggregates not only activate the transcription of NLRP3 inflammasome-related factors through the PPR-mediated NF-κB signaling pathway but also promote the assembly of the NLRP3 inflammasome due to mitochondrial dysfunction induced by α-syn PFFs [65, 69]. Blocking inflammasome activation with the inflammasome inhibitor MCC950 inhibits α-syn aggregate-induced secretion of IL-1β and improves motor behavior in a PFF mouse model [61]. In addition to membrane receptors, the membrane-associated intracellular tyrosine kinase Fyn also regulates α-syn PFF uptake into microglia, which results in oxidative stress due to mitochondrial damage by α-syn, contributing to inflammasome activation [65]. Compared to the activation of inflammasomes by a combination of LPS with ATP treatment [65], α-syn PFFs can induce both the priming and the activation of inflammasomes, which is closer to the activation of microglia under pathological conditions, further indicating that the microglial inflammation caused by α-syn aggregates involves multi-step processes (Fig. 1).

### Failure of Microglial Phagocytosis and Degradation Contributes to α-Syn Spreading

Microglia, as immune cells in the CNS, play a crucial role in the recognition and degradation of extracellular materials in the brain by phagocytosis [55]. In the CNS, phagocytosis is involved in the clearance of myelin debris, dead cells, and protein aggregates, as well as the pruning of synapses. Dysfunction of microglial phagocytosis disrupts brain homeostasis and leads to neurological disorders [55]. Phagocytosis is involved in the recognition of targets by the appropriate receptors on the cell membrane, the formation of phagosomes though membrane extension mediated by actin polymerization, and the transport of phagosomes to lysosomes for degradation [74, 75]. Some phagocytic components involved in the formation and transport of phagosomes to lysosomes overlap with components involved in autophagy [76–78]. For example, the formation of autophagic vesicles acquires microtubule-associated protein 1A/1B-light chain 3, which is also responsible for the maturation of phagocytic vesicles [78]. The impairment of microglial phagocytic function is closely related to aging [79, 80]. During aging, microglia increase the production of inflammatory factors and decrease phagocytosis, which contribute to aging-related diseases, such as AD and PD [79, 80]. The involvement of microglial phagocytosis in the processing of extracellular Aβ aggregates has been well documented in AD [79, 81, 82]. Using single-cell sequencing analyses, a presumably protective phagocytic microglial population in an AD mouse model has been identified, called disease-associated microglia (DAM) [82]. DAM activation requires the downregulation of homeostatic checkpoints and the initiation of a TREM2-dependent signaling pathway that enhances the cellular phagocytosis and degradation [82]. Loss of TREM2 leads to an exacerbation of Aβ pathology in an AD mouse model [82, 83], demonstrating the important roles of microglial phagocytosis in neurodegenerative diseases.

The involvement of microglial phagocytosis in α-syn pathology is supported by a recent study showing that microglia phagocytose and degrade α-syn aggregates by the redistribution of fibrillar α-syn through intercellular connections [84]. Under physiological conditions, microglia clear α-syn fibrils by the phagocytosis of extracellular α-syn aggregates into cells and the transport of α-syn aggregates into lysosomes for degradation, which prevents the spread of α-syn. The transfer of α-syn fibrils from activated microglia to the surrounding naïve microglia promotes the degradation of α-syn aggregates and decreases the inflammatory activity in α-syn-overloaded microglia [84]. IL-4 secreted from mesenchymal stem cells can modulate M2 microglial polarization, which promotes the phagocytosis and degradation of α-syn *in vitro* and *in vivo* and has anti-inflammatory effects [85, 86]. In a mouse model, α-syn released by neurons is degraded by selective autophagy after the endocytosis of α-syn by microglia, a process that is TLR4-dependent [87]. Moreover, α-syn accumulates to form high molecular weight species at 6 weeks after AAV-α-syn injection into mice in which microglial autophagy is deficient but not in WT mice [87]. Thus, the data support the hypothesis that microglia play a beneficial role in restricting α-syn accumulation and spread by the phagocytosis and degradation of α-syn aggregates released from neurons under α-syn transmission.

Although microglia can block α-syn spreading by the phagocytosis and degradation of extracellular α-syn aggregates under physiological conditions, the capacity for α-syn clearance by microglia declines under pathological conditions or aging [79]. With aging, microglial phagocytosis is impaired, resulting in a decrease in α-syn phagocytosis and an increase in α-syn accumulation in the brain [79, 88–90]. Microglia isolated from adult mice show decreased phagocytosis of α-syn oligomers compared with those isolated from young mice, and these microglia release more inflammatory cytokines [91]. Consistent with this conclusion, upregulation of CD22 expression is closely associated with age-related decrease in microglial phagocytosis. Anti-CD22 treatment increases the phagocytosis of pathological α-syn fibrils in microglia [79]. Moreover, α-syn aggregates interfere with the phagocytosis of microglia through the activation of SHP-1 [88], a negative regulator of phagocytosis [92]. Furthermore, autophagy proteins are decreased during aging, which directly affects the autophagic clearance of α-syn [87, 89]. Although microglial phagocytosis is generally enhanced when microglia are activated, continuous inflammation may impair this process [64, 80]. Continuous activation of microglia by α-syn aggravates inflammasome activation and IL-1β release, while inhibition of inflammasomal activation increases the phagocytosis and degradation of α-syn oligomers [64], indicating that inflammasome activation impairs the microglial phagocytosis and degradation of α-syn. In line with this finding, the transmission of α-syn pathology from the striatum to the SNpc region is significantly reduced in mice that receive a single injection of PFFs into the striatum if the mice are treated with the NLRP3 inflammasome inhibitor MCC950 [61]. Therefore, the impairment of microglial phagocytosis and degradation caused by pathological factors and aging accelerates the transmission of α-syn pathology (Fig. 1).

### Acceleration of α-Syn Spreading by Neuroinflammation

The evidence that neuroinflammation is linked to α-syn pathology in the human PD brain comes from a study by Olanow and colleagues. They showed that there are many DA neurons at 18 months after transplantation of fetal mesencephalic tissue into the striatum of PD patients, and only diffuse monomeric but not aggregated α-syn is present in the grafts until 14–16 years after transplantation. However, activated microglia are present in all grafts between 18 months and 16 years, much earlier than α-syn aggregation, suggesting that the activation of microglia plays roles in α-syn pathology [93]. This finding has been supported by a study in which the effects of microglia on α-syn transmission were evaluated in a mouse model [86]. In this model, the animals first received an injection of AAV virus that expressed human α-syn into the SN, followed by an intrastriatal injection of LPS two weeks later, and the animals then received an intrastriatal injection of healthy mouse embryonic DA neurons one week after LPS treatment. Human α-syn in TH-positive grafted DA neurons was significantly increased in animals treated with LPS, suggesting that inflammation promotes the cell-to-cell transmission of α-syn [86]. The cell-to-cell transmission of α-syn also increases when using a colony stimulating factor 1 receptor inhibitor to remove microglia in the brain [86]. This study demonstrates that microglia are involved in the clearance of α-syn; however, the activation of microglia under pathological conditions promotes the spread of α-syn pathology.

Activated microglia can secrete a wide range of inflammatory factors or exosomes into the extracellular environment and act on other cells, playing a key role in the communication between microglia and other cells [94]. The pro-inflammatory cytokines IL-1β and tumor necrosis factor α (TNF-α) that are released by microglia or other immune cells induce NF-κB activation in neurons, which is able to upregulate α-syn gene transcription through the recruitment of both the NF-κB subunits p65 and p50 and the cofactor p300 to the α-syn gene promoter, suggesting that α-syn-mediated neuroinflammation in turn accelerates α-syn transmission through an increase in α-syn expression in neurons [95]. Aging microglia have been shown to have increase inflammatory responses, releasing various inflammatory factors that may also affect α-syn expression in neurons. It has been reported that microglia treated with iron show certain characteristics of senescence. Treatment of neurons with conditioned media from these aging microglia also increases α-syn expression and aggregation [96]. Primary microglia treated with PFFs phagocytose α-syn aggregates and secrete α-syn oligomers *via* exosomes. Activation of microglia by LPS significantly increases exosome release after PFF treatment [97]. Treatment of primary neurons with microglia-derived exosomes induces α-syn aggregation in neurons that is more severe in combination with cytokines. Injection of exosomes from PFF-treated microglia or from the cerebrospinal fluid of PD patients into mouse brains induces α-syn aggregate formation in neurons, further suggesting that exosomes from microglia contribute to the transmission of α-syn pathology [97]. Thus, microglia promote α-syn transmission in multiple ways, either by the reduction of α-syn endocytosis and increase of the release of α-syn in microglia or by the induction of α-syn transmission between neurons, which contributes to the spread of α-syn pathology in the CNS (Fig. 1).

## Astrocytes and α-Syn Pathology

As the most abundant cell population in the CNS, astrocytes perform a range of actions to maintain brain function and homeostasis, including blood–brain barrier formation and maintenance, neurotransmitter transmission, regulation of synaptic plasticity and brain metabolism, and neuroimmunity [98–100]. Impairment of these actions lead to various neurological disorders and neurodegeneration [98, 99]. Postmortem and clinical studies have shown that astrocytes play a crucial role in the α-syn pathology in PD [101]. α-Syn aggregates released from neurons during α-syn transmission not only induce microglial activation but also activate astrocytes to exacerbate inflammation [102]. In postmortem PD brains, α-syn aggregates are present not only in DA neurons but also in astrocytes [101, 103]. Under physiological conditions, α-syn is rarely expressed or expressed at lower levels in astrocytes [101]. Therefore, the α-syn aggregates in astrocytes are believed to originate from neurons.

## Astrocyte-associated Neuroinflammation Caused by α-Syn Species

The first experimental evidence demonstrating that α-syn oligomers are transferred from neurons to astrocytes and subsequently activate astrocytes came from the study by He-Jin *et al.* [102]. In a co-culture system in which differentiated SH-SY5Y cells that overexpress α-syn are co-cultured with primary astrocytes, neuron-derived α-syn oligomers can be transferred to astrocytes [102]. In transgenic mice that overexpress human α-syn under the control of neuronal promoters, α-syn aggregates form in astrocytes, suggesting the transmission of α-syn from neurons to astrocytes [102]. The endocytosis of neuron-derived α-syn oligomers by astrocytes results in the accumulation of α-syn in astrocytes, leading to an inflammatory response of astrocytes, which enhances inflammatory cytokine production by astrocytes. Moreover, α-syn is co-localized with LAMP2, a lysosomal protein in astrocytes. Furthermore, the inhibition of lysosomes by bafilomycin A1, a lysosomal inhibitor, increases the accumulation of detergent-insoluble α-syn in astrocytes, suggesting that endocytic α-syn oligomers undergo lysosomal degradation. Importantly, the secretion of pro-inflammatory factors by astrocytes is dramatically increased in bafilomycin A1-treated astrocytes, demonstrating a correlation between the increased inflammatory response and the accumulation of α-syn in astrocytes [102].

Many molecules are involved in the α-syn aggregate-mediated activation of astrocytes [63, 104]. TLR4 is involved in α-syn oligomer-mediated astrocyte activation [63]. In human primary neurons, blockade of TLR4 does not affect α-syn oligomer-induced neuronal death. However, in a co-culture of human primary neurons and astrocytes, TLR4 receptor antagonists decrease the TNF-α levels and neuronal death induced by α-syn oligomers, suggesting that TLR4-mediated astrocyte activation by α-syn promotes neurodegeneration [63]. Moreover, NF-κB signaling is involved in the PFF-induced activation of astrocytes [104]. The expression and nuclear translocation of NF-κB are increased in human primary astrocytes after overnight treatment with PFFs [104]. Inhibition of NF-κB signaling with BAY, which inhibits the NF-κB upstream kinase inhibitor of IκB kinase, blocks the PFF-induced activation of NF-κB signaling and the production of inflammatory chemokines. Meanwhile, BAY also downregulates the gene expression profiles of A1 astrocytes, a neurotoxic state, and upregulates the gene expression of A2 astrocytes, a neurotrophic state, in PFF-treated astrocytes [104]. In addition, inhibition of receptor interacting protein kinase (RIPK) signaling with either RIPK3 or RIPK1 blocks NF-κB-associated gene expression and decreases chemokine CXCL10 levels in PFF-treated astrocytes, suggesting an involvement of RIPK signaling in PFF-induced astrocyte activation. In addition to activation of the pro-inflammatory response, PFFs impair the phagocytic activity of astrocytes, as evidenced by decreases in the expression of the phagocytosis-associated genes *GAS6* and *MEGF10* and in the uptake of fluorescently-labeled zymosan, an indicator of endocytosis [104].

In addition to direct activation of astrocytes by α-syn aggregates, microglia–astrocyte communications are also important in PFF-induced astrocyte activation [60]. Pro-inflammatory factors that are released by activated microglia upon PFF stimulation can convert the remaining astrocytes to the A1 type, which is neurotoxic. Three key inflammatory mediators, TNF-α, IL-1α, and complement component 1q, which are produced by activated microglia, contribute to inflammatory communication between microglia and astrocytes. Furthermore, the glucagon-like peptide-1 receptor agonist NLY01 can inhibit the activated microglia-induced conversion of astrocytes to the neurotoxic A1 type, prevent DA neuronal loss, and improve behavioral deficits in the PFF mouse model [60]. Thus, data suggest that α-syn aggregates induce inflammation in the brain through direct or indirect pathways that involve both microglia and astrocytes, and this contributes to the neurodegeneration in PD (Fig. 1).

### Astrocytes as Modulators of α-Syn Spreading

Lines of evidence suggest that astrocytes can endocytose neuron-derived α-syn aggregates and transport them to lysosomes for degradation, similar to the process in microglia [102, 105, 106]. Astrocytes degrade α-syn aggregates more effectively than neurons, which may be attributed to the higher abundance of lysosomes in astrocytes [105]. By co-culturing astrocytes with iPSC-derived DA neurons carrying *SNCA* triplications, astrocytes reduce α-syn aggregation in neurons and α-syn transmission between neurons, suggesting that astrocytes have protective effects to limit α-syn transmission. However, astrocytes with an *ATP13A2* mutation lose the capacity for endocytosis and degradation of α-syn aggregates that are released from neurons, resulting in an increase in α-syn transmission, suggesting that functional impairment of astrocytes might accelerate α-syn transmission [105]. Moreover, an increase in mitochondrial fragmentation and a decrease in ATP production has been reported in astrocytes treated with α-syn oligomers, suggesting that an overload of α-syn aggregates in astrocytes affects the normal function of astrocytes, contributing to the loss of a protective role in the inhibition of α-syn transmission [107, 108]. In addition, α-syn transmission between astrocytes has also been reported [108]. Similar to the manner in which microglia distribute α-syn PFFs through an intercellular network, astrocytes can transfer α-syn oligomers to nearby astrocytes *via* the formation of tunneling nanotubes (TNTs) [108]. The accumulation of α-syn in astrocytes induces the formation of TNTs to transfer α-syn to nearby astrocytes. Moreover, α-syn oligomers lead to morphological alterations in the endoplasmic reticulum and mitochondria. Furthermore, the accumulation of α-syn impairs autophagic flux, as evidenced by an increase in the formation of autophagosomes that are not degraded by lysosomes [108].

## Conclusions and Perspectives

**Fig. 2:**
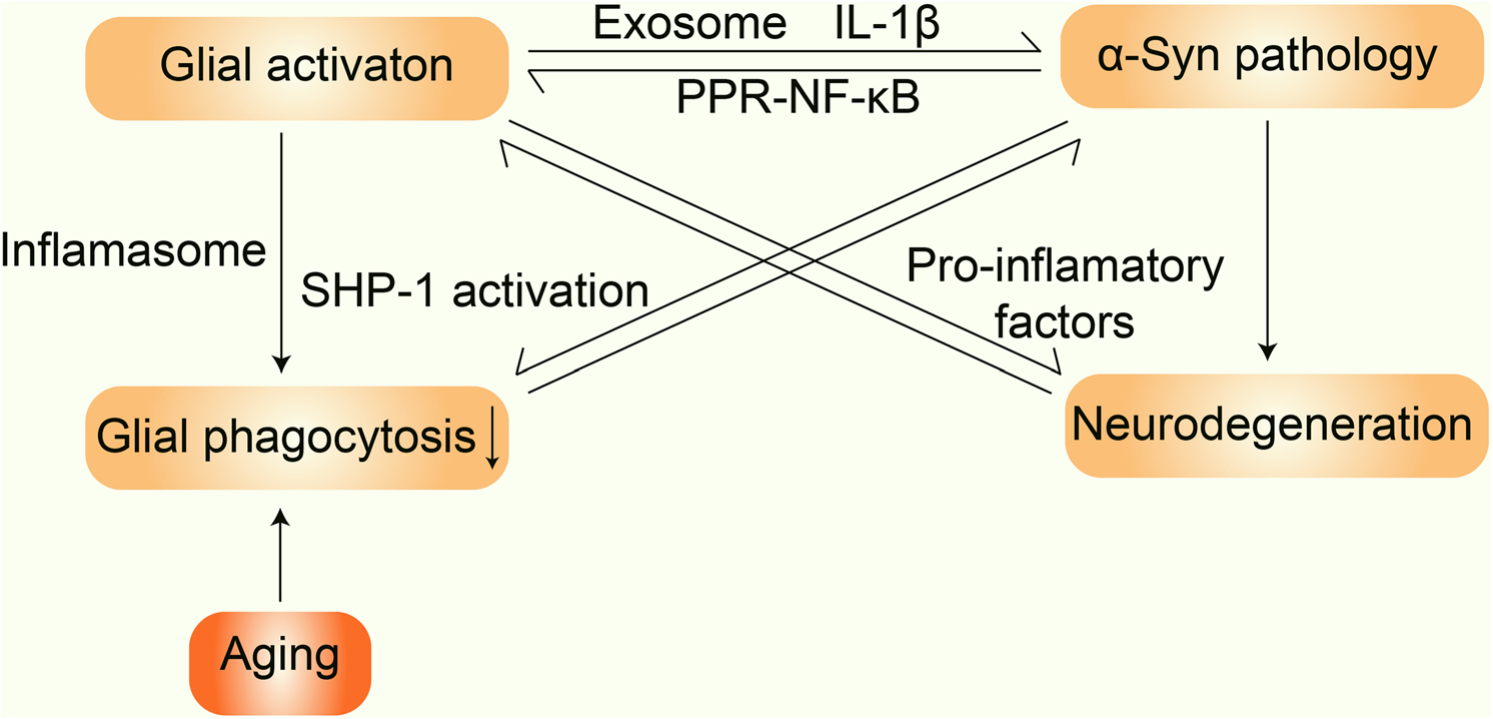
Schematic of the interactions among glial activation, α-syn pathology, and neurodegeneration in PD. α-Syn pathology leads to the activation of glia, which contributes to α-syn pathology. Neurodegeneration occurs due to either the direct neurotoxicity of α-syn aggregates or the release of pro-inflammatory factors by the activated glia. In addition, glial activation and α-syn aggregation as well as aging lead to a decrease in phagocytosis by glial cells, resulting in an accelerated spread of α-syn pathology.

There is a link between glial activation and α-syn pathology in PD pathogenesis. On the one hand, the glial activation response to α-syn oligomers, at least at the early stage, promotes the phagocytosis and clearance of α-syn by glia, which inhibits the transmission of α-syn between neurons and the development of α-syn pathology. On the other hand, sustained activation of glial cells by α-syn aggregates leads to chronic inflammation, which impairs the phagocytic activity of glia and increases inflammatory cytokine levels, leading to the accumulation of α-syn and increases in the cell-to-cell transmission of α-syn, contributing to the spread of α-syn pathology. In addition, genetic and environmental factors as well as aging influence both glia and neurons, and are involved in the initiation and progression of α-syn pathology (Fig. 2). Drugs that modulate the microglial and astrocyte activation associated with α-syn alleviate the loss of DA neurons and the defects in behaviors in PFF mouse models, further demonstrating the pivotal roles of α-syn-mediated glial activation in PD pathogenesis. However, some questions remain to be elucidated. Do temporal and spatial glial activation contribute to the spread and distribution of α-syn pathology in PD and other α-syn-related neurodegenerative diseases? Why do different neurons have differential responses and susceptibilities to α-syn oligomers, although the transmission of α-syn pathology still occurs within them? In future, the identification of factors with analyses using spatial transcriptomics and single-cell sequencing in PD animal models may reveal a mechanistic connection between microglial activation and α-syn pathology. Furthermore, more microglial and astrocyte subtypes, in addition to M1, M2, A1, and A2, can be identified in PD models using single-cell transcriptomics, which may detail the roles of the subtypes of glial cells in the clearance and release of α-syn. This would also be helpful for exploring the roles of neurons in response to α-syn species and in the spread of α-syn pathology.

## References

[1] Li S, Jia C, Li T, Le W (2021). Hot topics in recent Parkinson's disease research: Where we are and where we should go. Neurosci Bull.

[2] Corti O, Lesage S, Brice A (2011). What genetics tells us about the causes and mechanisms of Parkinson's disease. Physiol Rev.

[3] Deng H, Wang P, Jankovic J (2018). The genetics of Parkinson disease. Ageing Res Rev.

[4] Marras C, Beck JC, Bower JH, Roberts E, Ritz B, Ross GW (2018). Prevalence of Parkinson's disease across north *America*. Npj Park Dis.

[5] Wang R, Sun H, Ren H, Wang G (2020). α-Synuclein aggregation and transmission in Parkinson's disease: A link to mitochondria and lysosome. Sci China Life Sci.

[6] Kalia LV, Lang AE (2015). Parkinson's disease. Lancet.

[7] Goedert M, Spillantini MG, del Tredici K, Braak H (2013). 100 years of Lewy pathology. Nat Rev Neurol.

[8] Wong YC, Krainc D (2017). α-synuclein toxicity in neurodegeneration: Mechanism and therapeutic strategies. Nat Med.

[9] Wang X, Wang R, Li J (2022). Influence of sleep disruption on protein accumulation in neurodegenerative diseases. Ageing Neur Dis.

[10] Shahmoradian SH, Lewis AJ, Genoud C, Hench J, Moors TE, Navarro PP (2019). Lewy pathology in Parkinson's disease consists of crowded organelles and lipid membranes. Nat Neurosci.

[11] Spillantini MG, Crowther RA, Jakes R, Hasegawa M, Goedert M (1998). Alpha-Synuclein in filamentous inclusions of Lewy bodies from Parkinson's disease and dementia with lewy bodies. Proc Natl Acad Sci U S A.

[12] Iwatsubo T, Yamaguchi H, Fujimuro M, Yokosawa H, Ihara Y, Trojanowski JQ (1996). Purification and characterization of Lewy bodies from the brains of patients with diffuse Lewy body disease. Am J Pathol.

[13] Gould N, Mor DE, Lightfoot R, Malkus K, Giasson B, Ischiropoulos H (2014). Evidence of native α-synuclein conformers in the human brain. J Biol Chem.

[14] Wang W, Perovic I, Chittuluru J, Kaganovich A, Nguyen LTT, Liao J (2011). A soluble α-synuclein construct forms a dynamic tetramer. Proc Natl Acad Sci U S A.

[15] Bartels T, Choi JG, Selkoe DJ (2011). α-Synuclein occurs physiologically as a helically folded tetramer that resists aggregation. Nature.

[16] Kustrimovic N, Marino F, Cosentino M (2019). Peripheral immunity, immunoaging and neuroinflammation in Parkinson's disease. Curr Med Chem.

[17] Pajares M, Rojo AI, Manda G, Boscá L, Cuadrado A (2020). Inflammation in Parkinson's disease: Mechanisms and therapeutic implications. Cells.

[18] Tansey MG, Wallings RL, Houser MC, Herrick MK, Keating CE, Joers V. Inflammation and immune dysfunction in Parkinson disease. Nat Rev Immunol 2022: 1–17. doi: 10.1038/s41577-022-00684-6. 10.1038/s41577-022-00684-6 PMC8895080 35246670

[19] Dickson DW (2012). Parkinson's disease and Parkinsonism: Neuropathology. Cold Spring Harb Perspect Med.

[20] Bartels T, De Schepper S, Hong S (2020). Microglia modulate neurodegeneration in Alzheimer's and Parkinson's diseases. Science.

[21] Spillantini MG, Schmidt ML, Lee VMY, Trojanowski JQ, Jakes R, Goedert M (1997). α-synuclein in lewy bodies. Nature.

[22] Burré J, Sharma M, Tsetsenis T, Buchman V, Etherton MR, Südhof TC (2010). Alpha-synuclein promotes SNARE-complex assembly *in vivo* and *in vitro*. Science.

[23] Bendor JT, Logan TP, Edwards RH (2013). The function of α-synuclein. Neuron.

[24] Cremades N, Cohen SIA, Deas E, Abramov AY, Chen AY, Orte A (2012). Direct observation of the interconversion of normal and toxic forms of α-synuclein. Cell.

[25] Soto C, Pritzkow S (2018). Protein misfolding, aggregation, and conformational strains in neurodegenerative diseases. Nat Neurosci.

[26] Jucker M, Walker LC (2013). Self-propagation of pathogenic protein aggregates in neurodegenerative diseases. Nature.

[27] Winner B, Jappelli R, Maji SK, Desplats PA, Boyer L, Aigner S (2011). *In vivo* demonstration that alpha-synuclein oligomers are toxic. Proc Natl Acad Sci U S A.

[28] Rockenstein E, Nuber S, Overk CR, Ubhi K, Mante M, Patrick C (2014). Accumulation of oligomer-prone α-synuclein exacerbates synaptic and neuronal degeneration *in vivo*. Brain.

[29] Prots I, Grosch J, Brazdis RM, Simmnacher K, Veber V, Havlicek S (2018). α-Synuclein oligomers induce early axonal dysfunction in human iPSC-based models of synucleinopathies. Proc Natl Acad Sci U S A.

[30] Rocha S, Kumar R, Nordén B, Wittung-Stafshede P (2021). Orientation of α-synuclein at negatively charged lipid vesicles: Linear dichroism reveals time-dependent changes in *Helix* binding mode. J Am Chem Soc.

[31] Antonschmidt L, Dervişoğlu R, Sant V, Tekwani Movellan K, Mey I, Riedel D (2021). Insights into the molecular mechanism of amyloid filament formation: Segmental folding of α-synuclein on lipid membranes. Sci Adv.

[32] Fusco G, Chen SW, Williamson PTF, Cascella R, Perni M, Jarvis JA (2017). Structural basis of membrane disruption and cellular toxicity by α-synuclein oligomers. Science.

[33] Moors TE, Maat CA, Niedieker D, Mona D, Petersen D, Timmermans-Huisman E (2021). The subcellular arrangement of alpha-synuclein proteoforms in the Parkinson's disease brain as revealed by multicolor STED microscopy. Acta Neuropathol.

[34] Cascella R, Chen SW, Bigi A, Camino JD, Xu CK, Dobson CM (1814). The release of toxic oligomers from α-synuclein fibrils induces dysfunction in neuronal cells. Nat Commun.

[35] Biswas B, Roy S, Mondal JA, Singh PC (2020). Interaction of α-synuclein with phospholipids and the associated restructuring of interfacial lipid water: An interface-selective vibrational spectroscopic study. Angew Chem Int Ed Engl.

[36] Maio RD, Barrett PJ, Hoffman EK, Barrett CW, Zharikov A, Borah A (2016). α-Synuclein binds to TOM20 and inhibits mitochondrial protein import in Parkinson's disease. Sci Transl Med.

[37] Ludtmann MHR, Angelova PR, Horrocks MH, Choi ML, Rodrigues M, Baev AY (2018). α-synuclein oligomers interact with ATP synthase and open the permeability transition pore in Parkinson's disease. Nat Commun.

[38] De Miranda BR, Rocha EM, Castro SL, Greenamyre JT (2020). Protection from α-Synuclein induced dopaminergic neurodegeneration by overexpression of the mitochondrial import receptor TOM20. NPJ Parkinsons Dis.

[39] Braak H, Ghebremedhin E, Rüb U, Bratzke H, Del Tredici K (2004). Stages in the development of Parkinson's disease-related pathology. Cell Tissue Res.

[40] Braak H, Tredici KD, Rüb U, de Vos RAI, Jansen Steur ENH, Braak E (2003). Staging of brain pathology related to sporadic Parkinson's disease. Neurobiol Aging.

[41] Kordower JH, Chu Y, Hauser RA, Freeman TB, Olanow CW (2008). Lewy body–like pathology in long-term embryonic nigral transplants in Parkinson's disease. Nat Med.

[42] Li JY, Englund E, Holton JL, Soulet D, Hagell P, Lees AJ (2008). Lewy bodies in grafted neurons in subjects with Parkinson's disease suggest host-to-graft disease propagation. Nat Med.

[43] Luk KC, Song C, O'Brien P, Stieber A, Branch JR, Brunden KR (2009). Exogenous alpha-synuclein fibrils seed the formation of Lewy body-like intracellular inclusions in cultured cells. Proc Natl Acad Sci U S A.

[44] Volpicelli-Daley LA, Luk KC, Patel TP, Tanik SA, Riddle DM, Stieber A (2011). Exogenous α-synuclein fibrils induce lewy body pathology leading to synaptic dysfunction and neuron death. Neuron.

[45] Mahul-Mellier AL, Burtscher J, Maharjan N, Weerens L, Croisier M, Kuttler F (2020). The process of Lewy body formation, rather than simply α-synuclein fibrillization, is one of the major drivers of neurodegeneration. Proc Natl Acad Sci U S A.

[46] Luk KC, Kehm VM, Zhang B, O'Brien P, Trojanowski JQ, Lee VMY (2012). Intracerebral inoculation of pathological α-synuclein initiates a rapidly progressive neurodegenerative α-synucleinopathy in mice. J Exp Med.

[47] Luk KC, Kehm V, Carroll J, Zhang B, O’Brien P, Trojanowski JQ (2012). Pathological α-synuclein transmission initiates parkinson-like neurodegeneration in nontransgenic mice. Science.

[48] Vargas JY, Grudina C, Zurzolo C (2019). The prion-like spreading of α-synuclein: From *in vitro* to *in vivo* models of Parkinson's disease. Ageing Res Rev.

[49] Volpicelli-Daley LA, Luk KC, Lee VMY (2014). Addition of exogenous α-synuclein preformed fibrils to primary neuronal cultures to seed recruitment of endogenous α-synuclein to Lewy body and Lewy neurite–like aggregates. Nat Protoc.

[50] Nonaka T, Watanabe ST, Iwatsubo T, Hasegawa M (2010). Seeded aggregation and toxicity of{alpha}-synuclein and tau: Cellular models of neurodegenerative diseases. J Biol Chem.

[51] Luk KC, Kehm V, Carroll J, Zhang B, O'Brien P, Trojanowski JQ (2012). Pathological α-synuclein transmission initiates Parkinson-like neurodegeneration in nontransgenic mice. Science.

[52] Challis C, Hori A, Sampson TR, Yoo BB, Challis RC, Hamilton AM (2020). Gut-seeded α-synuclein fibrils promote gut dysfunction and brain pathology specifically in aged mice. Nat Neurosci.

[53] Kim S, Kwon SH, Kam TI, Panicker N, Karuppagounder SS, Lee S (2019). Transneuronal propagation of pathologic α-synuclein from the gut to the brain models Parkinson's disease. Neuron.

[54] Stowell RD, Sipe GO, Dawes RP, Batchelor HN, Lordy KA, Whitelaw BS (2019). Noradrenergic signaling in the wakeful state inhibits microglial surveillance and synaptic plasticity in the mouse visual cortex. Nat Neurosci.

[55] Colonna M, Butovsky O (2017). Microglia function in the central nervous system during health and neurodegeneration. Annu Rev Immunol.

[56] Wang X (2021). A bridge between the innate immunity system and amyloid-β production in Alzheimer's disease. Neurosci Bull.

[57] McGeer PL, Itagaki S, Boyes BE, McGeer EG (1988). Reactive microglia are positive for HLA-DR in the substantia nigra of Parkinson's and Alzheimer's disease brains. Neurology.

[58] Yu YX, Li YP, Gao F, Hu QS, Zhang Y, Chen D (2016). Vitamin K2 suppresses rotenone-induced microglial activation in vitro. Acta Pharmacol Sin.

[59] Song N, Chen L, Xie J (2021). Alpha-synuclein handling by microglia: Activating, combating, and worsening. Neurosci Bull.

[60] Yun SP, Kam TI, Panicker N, Kim S, Oh Y, Park JS (2018). Block of A1 astrocyte conversion by microglia is neuroprotective in models of Parkinson's disease. Nat Med.

[61] Gordon R, Albornoz EA, Christie DC, Langley MR, Kumar V, Mantovani S, *et al*. Inflammasome inhibition prevents α-synuclein pathology and dopaminergic neurodegeneration in mice. Sci Transl Med 2018, 10: eaah4066. 10.1126/scitranslmed.aah4066 PMC6483075 30381407

[62] Kim C, Ho DH, Suk JE, You S, Michael S, Kang J (2013). Neuron-released oligomeric α-synuclein is an endogenous agonist of TLR2 for paracrine activation of microglia. Nat Commun.

[63] Hughes CD, Choi ML, Ryten M, Hopkins L, Drews A, Botía JA (2019). Picomolar concentrations of oligomeric alpha-synuclein sensitizes TLR4 to play an initiating role in Parkinson's disease pathogenesis. Acta Neuropathol.

[64] Scheiblich H, Bousset L, Schwartz S, Griep A, Latz E, Melki R (2021). Microglial NLRP3 inflammasome activation upon TLR2 and TLR5 ligation by distinct α-synuclein assemblies. J Immunol.

[65] Panicker N, Sarkar S, Harischandra DS, Neal M, Kam TI, Jin H (2019). Fyn kinase regulates misfolded α-synuclein uptake and NLRP3 inflammasome activation in microglia. J Exp Med.

[66] Stefanova N, Fellner L, Reindl M, Masliah E, Poewe W, Wenning GK (2011). Toll-like receptor 4 promotes α-synuclein clearance and survival of nigral dopaminergic neurons. Am J Pathol.

[67] Zhang W, Wang T, Pei Z, Miller DS, Wu X, Block ML (2005). Aggregated alpha-synuclein activates microglia: A process leading to disease progression in Parkinson's disease. FASEB J.

[68] Cao S, Theodore S, Standaert DG (2010). Fcγ receptors are required for NF-κB signaling, microglial activation and dopaminergic neurodegeneration in an AAV-synuclein mouse model of Parkinson's disease. Mol Neurodegener.

[69] Pike AF, Varanita T, Herrebout MAC, Plug BC, Kole J, Musters RJP (2021). α-Synuclein evokes NLRP3 inflammasome-mediated IL-1β secretion from primary human microglia. Glia.

[70] Broz P, Dixit VM (2016). Inflammasomes: Mechanism of assembly, regulation and signalling. Nat Rev Immunol.

[71] Swanson KV, Deng M, Ting JPY (2019). The NLRP3 inflammasome: Molecular activation and regulation to therapeutics. Nat Rev Immunol.

[72] Halle A, Hornung V, Petzold GC, Stewart CR, Monks BG, Reinheckel T (2008). The NALP3 inflammasome is involved in the innate immune response to amyloid-β. Nat Immunol.

[73] Ising C, Venegas C, Zhang S, Scheiblich H, Schmidt SV, Vieira-Saecker A (2019). NLRP3 inflammasome activation drives tau pathology. Nature.

[74] Caron E, Hall A (1998). Identification of two distinct mechanisms of phagocytosis controlled by different Rho GTPases. Science.

[75] Tremblay ME, Cookson MR, Civiero L (2019). Glial phagocytic clearance in Parkinson's disease. Mol Neurodegeneration.

[76] Münz C (2017). Autophagy proteins in phagocyte endocytosis and exocytosis. Front Immunol.

[77] Sanjuan MA, Dillon CP, Tait SWG, Moshiach S, Dorsey F, Connell S (2007). Toll-like receptor signalling in macrophages links the autophagy pathway to phagocytosis. Nature.

[78] Martinez J, Almendinger J, Oberst A, Ness R, Dillon CP, Fitzgerald P (2011). Microtubule-associated protein 1 light chain 3 alpha (LC3)-associated phagocytosis is required for the efficient clearance of dead cells. Proc Natl Acad Sci U S A.

[79] Pluvinage JV, Haney MS, Smith BAH, Sun J, Iram T, Bonanno L (2019). CD22 blockade restores homeostatic microglial phagocytosis in ageing brains. Nature.

[80] Marschallinger J, Iram T, Zardeneta M, Lee SE, Lehallier B, Haney MS (2020). Lipid-droplet-accumulating microglia represent a dysfunctional and proinflammatory state in the aging brain. Nat Neurosci.

[81] Grubman A, Choo XY, Chew G, Ouyang JF, Sun G, Croft NP (2021). Transcriptional signature in microglia associated with Aβ plaque phagocytosis. Nat Commun.

[82] Keren-Shaul H, Spinrad A, Weiner A, Matcovitch-Natan O, Dvir-Szternfeld R, Ulland TK (2017). A unique microglia type associated with restricting development of Alzheimer's disease. Cell.

[83] Gratuze M, Chen Y, Parhizkar S, Jain N, Strickland MR, Serrano JR (2021). Activated microglia mitigate Aβ-associated tau seeding and spreading. J Exp Med.

[84] Scheiblich H, Dansokho C, Mercan D, Schmidt SV, Bousset L, Wischhof L (2021). Microglia jointly degrade fibrillar alpha-synuclein cargo by distribution through tunneling nanotubes. Cell.

[85] Park HJ, Oh SH, Kim HN, Jung YJ, Lee PH (2016). Mesenchymal stem cells enhance α-synuclein clearance via M2 microglia polarization in experimental and human parkinsonian disorder. Acta Neuropathol.

[86] George S, Rey NL, Tyson T, Esquibel C, Meyerdirk L, Schulz E (2019). Microglia affect α-synuclein cell-to-cell transfer in a mouse model of Parkinson's disease. Mol Neurodegener.

[87] Choi I, Zhang Y, Seegobin SP, Pruvost M, Wang Q, Purtell K (2020). Microglia clear neuron-released α-synuclein via selective autophagy and prevent neurodegeneration. Nat Commun.

[88] Choi YR, Kang SJ, Kim JM, Lee SJ, Jou I, Joe EH (2015). FcγRIIB mediates the inhibitory effect of aggregated α-synuclein on microglial phagocytosis. Neurobiol Dis.

[89] Lipinski MM, Zheng B, Lu T, Yan Z, Py BF, Ng A (2010). Genome-wide analysis reveals mechanisms modulating autophagy in normal brain aging and in Alzheimer's disease. Proc Natl Acad Sci U S A.

[90] Tu HY, Yuan BS, Hou XO, Zhang XJ, Pei CS, Ma YT (2021). α-synuclein suppresses microglial autophagy and promotes neurodegeneration in a mouse model of Parkinson's disease. Aging Cell.

[91] Bliederhaeuser C, Grozdanov V, Speidel A, Zondler L, Ruf WP, Bayer H (2016). Age-dependent defects of alpha-synuclein oligomer uptake in microglia and monocytes. Acta Neuropathol.

[92] Scharenberg AM, Kinet JP (1996). The emerging field of receptor-mediated inhibitory signaling: SHP or SHIP?. Cell.

[93] Olanow CW, Savolainen M, Chu Y, Halliday GM, Kordower JH (2019). Temporal evolution of microglia and α-synuclein accumulation following foetal grafting in Parkinson's disease. Brain.

[94] Delpech JC, Herron S, Botros MB, Ikezu T (2019). Neuroimmune crosstalk through extracellular vesicles in health and disease. Trends Neurosci.

[95] Dutta D, Jana M, Majumder M, Mondal S, Roy A, Pahan K (2021). Selective targeting of the TLR2/MyD88/NF-κB pathway reduces α-synuclein spreading *in vitro* and *in vivo*. Nat Commun.

[96] Angelova DM, Brown DR (2018). Model senescent microglia induce disease related changes in α-synuclein expression and activity. Biomolecules.

[97] Guo M, Wang J, Zhao Y, Feng Y, Han S, Dong Q (2020). Microglial exosomes facilitate α-synuclein transmission in Parkinson's disease. Brain.

[98] Linnerbauer M, Wheeler MA, Quintana FJ (2020). Astrocyte crosstalk in CNS inflammation. Neuron.

[99] Giovannoni F, Quintana FJ (2020). The role of astrocytes in CNS inflammation. Trends Immunol.

[100] Escartin C, Galea E, Lakatos A, O’Callaghan JP, Petzold GC, Serrano-Pozo A (2021). Reactive astrocyte nomenclature, definitions, and future directions. Nat Neurosci.

[101] Braak H, Sastre M, Del Tredici K (2007). Development of α-synuclein immunoreactive astrocytes in the forebrain parallels stages of intraneuronal pathology in sporadic Parkinson's disease. Acta Neuropathol.

[102] Lee HJ, Suk JE, Patrick C, Bae EJ, Cho JH, Rho S (2010). Direct transfer of alpha-synuclein from neuron to astroglia causes inflammatory responses in synucleinopathies. J Biol Chem.

[103] Wakabayashi K, Hayashi S, Yoshimoto M, Kudo H, Takahashi H (2000). NACP/α-synuclein-positive filamentous inclusions in astrocytes and oligodendrocytes of Parkinson's disease brains. Acta Neuropathol.

[104] Chou TW, Chang NP, Krishnagiri M, Patel AP, Lindman M, Angel JP (2021). Fibrillar α-synuclein induces neurotoxic astrocyte activation via RIP kinase signaling and NF-κB. Cell Death Dis.

[105] Tsunemi T, Ishiguro Y, Yoroisaka A, Valdez C, Miyamoto K, Ishikawa K (2020). Astrocytes protect human dopaminergic neurons from α-synuclein accumulation and propagation. J Neurosci.

[106] Loria F, Vargas JY, Bousset L, Syan S, Salles A, Melki R (2017). α-Synuclein transfer between neurons and astrocytes indicates that astrocytes play a role in degradation rather than in spreading. Acta Neuropathol.

[107] Lindström V, Gustafsson G, Sanders LH, Howlett EH, Sigvardson J, Kasrayan A (2017). Extensive uptake of α-synuclein oligomers in astrocytes results in sustained intracellular deposits and mitochondrial damage. Mol Cell Neurosci.

[108] Rostami J, Holmqvist S, Lindström V, Sigvardson J, Westermark GT, Ingelsson M (2017). Human astrocytes transfer aggregated alpha-synuclein via tunneling nanotubes. J Neurosci.

[109] Daniele SG, Béraud D, Davenport C, Cheng K, Yin H, Maguire-Zeiss KA (2015). Activation of MyD88-dependent TLR1/2 signaling by misfolded α-synuclein, a protein linked to neurodegenerative disorders. Sci Signal.

[110] Fellner L, Irschick R, Schanda K, Reindl M, Klimaschewski L, Poewe W (2013). Toll-like receptor 4 is required for α-synuclein dependent activation of microglia and astroglia. Glia.

[111] Su X, Maguire-Zeiss KA, Giuliano R, Prifti L, Venkatesh K, Federoff HJ (2008). Synuclein activates microglia in a model of Parkinson's disease. Neurobiol Aging.

[112] Cao S, Standaert DG, Harms AS (2012). The gamma chain subunit of Fc receptors is required for alpha-synuclein-induced pro-inflammatory signaling in microglia. J Neuroinflammation.

